# The rodent aging interventions database (RAID): a data visualization tool for all studies reporting rodent lifespan extension

**DOI:** 10.18632/aging.206228

**Published:** 2025-03-27

**Authors:** Maximus V. Peto, Anthony J. Floyd, Ben Zealley, Aubrey D. N. J. de Grey

**Affiliations:** 1 Long Life Labs, LLC, Buffalo, WY 82834, USA; 2 Longevity Escape Velocity (LEV) Foundation, San Francisco, CA 94107, USA

**Keywords:** life extension, longevity, mice, rats, databases

## Abstract

Numerous studies have investigated the effects of various interventions on the lifespans of mice and rats. The design of future rodent lifespan extension experiments might consider experimental parameters used in earlier investigations, but finding and reviewing all previous experiments requires a substantial resource investment. Additionally, when studied collectively, the results of previous investigations might suggest fundamental mechanisms causing age-related degeneration. Here, we report our efforts to find and aggregate data from all research reports of lifespan extension in mice or rats, which we call the “Rodent Aging Interventions Database” (RAID). We identified studies for inclusion using complex PubMed queries and by nomination from our colleagues in the field. The relevant data from each study was manually extracted and recorded in a table. A publicly available, web-based software tool was then created to enable users to visualize and filter this data in a convenient manner. Our current dataset, covering publications up to October 2022, includes 121 unique studies reporting on 212 distinct intervention protocols that extended lifespan in mice or rats. We intend to periodically update our dataset as new rodent lifespan studies are reported. RAID is publicly available at https://levf.org/raid.

## INTRODUCTION

Rodent lifespan extension has been studied for decades. Complicating the analysis of this data is its heterogeneity regarding species, strain, sex, intervention type (genetic, dietary, pharmaceutical, etc.), age at administration, etc. We imagined several valuable outcomes—described below—which might result from aggregating this data and making it publicly available, easily navigable, and visually ranked according to the magnitude of lifespan extension reported.

### Enhance conceptual thinking about causes of age-related degeneration and mortality

Currently, there is no consensus among scientists about the ultimate causes of age-associated degeneration and increased mortality. Many different contributors to these phenomena have been explored, including accumulation of DNA damage [1], telomere shortening [2], accumulation of molecular and cellular damage in and around cells and tissues [3], a rise in the prevalence of senescent cells [4], genetic quasi-programs [5], and mitochondrial free radical generation [6], among others.

Causal theories of age-related degeneration and increased mortality must explain not only why these age-related phenomena occur but also why certain interventions (and not others) extend lifespan. If sufficient data about life-extending interventions can be accumulated and evaluated as a group, one might be better able to infer the underlying mechanisms by observing shared characteristics among interventions reported to extend lifespan.

For example, to the casual observer, several seemingly unrelated interventions have been reported to extend rodent lifespan, including knockout of type 5 adenylyl cyclase (AC5) [7], knockout of pregnancy-associated plasma protein A (PAPP-A) [8], and surgical removal of the pituitary gland [9]. However, each of these interventions were also reported to interfere with the growth hormone/insulin-like growth factor-1 (GH/IGF-1) signaling axis. Interference with this pathway has been noted to extend lifespan in earlier studies, such as was observed in Ames dwarf mice [10] and growth hormone receptor knockout (GHRKO) mice [11]. When viewed together, considering how GH/IGF-1 are related to growth during development [12], these results support the idea that events and circumstances associated with development might fundamentally affect an organism’s lifespan.

### Facilitate efficient planning of rodent life-extension experiments

When deciding on the parameters of a pending rodent life-extension investigation, it can be cumbersome to identify all literature relevant to the intervention(s) under consideration. For example, rapamycin has been reported to extend rodent lifespan in multiple studies, but the details of administration have varied and include 126 ppm in food [13], 14 ppm in food and combined with metformin [14], 42 ppm in food [15], 8 mg/kg injection [13], and 14.7 ppm in food with acarbose [16]. We designed our data visualization tool to be capable of quickly and easily searching for all studies using an intervention keyword. It is our hope that this tool will enable other researchers to more efficiently locate earlier findings which are relevant to their contemplated rodent lifespan experiments.

### Help clarify misunderstandings about rodent life-extension research

There appear to be significant misunderstandings about the state of life-extension research, some of which might be caused by inaccurate or misleading reporting on scientific studies. For example, the headline of an article by CNN Health on January 12^th^, 2023, read, “Old mice grow young again in study. Can people do the same?” [17]. The focus of that article was the report by [18] about the creation of a system the authors called “inducible changes to the epigenome” (ICE). This system was apparently created in part to evaluate the extent to which age-related epigenetic changes are associated with age-related alterations and to help corroborate the authors’ “information theory of aging”. Importantly, no magnitude of mouse lifespan extension was mentioned in the CNN article, even though the title of the article implies life extension by using the phase “old mice grow young again”. Sensational or hyperbolic titles of popular science articles might create misunderstandings about—and unrealistic expectations for—near-term prospects for human lifespan enhancement.

We believe that such misconceptions might be less frequent or more easily corrected given the existence of a reliable, manually verified, publicly available source of all scientific studies of successful rodent lifespan extension. Such a resource could help engender more accurate understanding about the current state of life-extension research and thereby facilitate more realistic expectations about the near-term prospects for human lifespan enhancement.

There have been multiple efforts to aggregate data about aging and lifespan in various animals. Among these include The Healthy Worm Database [19], which accumulates data on lifespan extension experiments in *Caenorhabditis elegans*. Another resource—Human Aging Genomic Resources (HAGR) [20]—includes multiple databases on topics such as animal life history data and molecular signatures of dietary restriction. And AgeMeta [21] is a database of mammalian gene expression during aging. However, we were unable to locate a comprehensive database of mouse and rat studies that reported statistically significant lifespan extension.

Motivated by the unique advantages that might be gained from a public database focused on rodent life extension, we have created the Rodent Aging Interventions Database (RAID)—a curated set of intervention studies reporting lifespan extension in rodents with an accompanying data visualization software tool that is publicly available on the Internet. We anticipate that RAID will enhance the ability of researchers to analyze the many studies of rodent lifespan extension and make inferences about the underlying mechanisms causing age-related degeneration and increased risk of mortality.

## RESULTS

Our PubMed queries used to find the studies to construct RAID resulted in a total of 1,677 studies to be evaluated for relevance according to their titles. Manual evaluation of these resulted in retaining 292 abstracts for subsequent evaluation of their manuscripts. The evaluation of those manuscripts, when combined with relevant nominations by authors and other colleagues, resulted in 121 unique studies being included in the final dataset, which included 212 distinct intervention protocols.

Due to variation between lifespans of control groups in different rodent experiments, the percent of lifespan extension reported can be misleading. For example, an extension of 10% in median lifespan relative to a control group with a median lifespan of 800 days is equivalent to a 13.3% lifespan extension relative to a control group with a median lifespan of 600 days (80 days of life extension for each). Therefore, in the RAID data visualization tool, lifespan extension is sorted in descending order according to the number of days of lifespan extension reported relative to controls, rather than the percentage of extension.

The mean or median (hereafter “mean/median”) lifespan extension in our dataset ranges from +18 days in male Fischer-344 rats administered 300 mg/kg/day metformin [22] to +930 days in male Wistar rats administered 1.7 mg/kg body weight of C60 fullerene in olive oil multiple times via oral gavage [23]. Maximum lifespan extension in our dataset ranges from +28 days in female UM-HET3 mice administered acarbose at 1,000 ppm in food [24] to +870 days in male Wistar rats administered 1.7 mg/kg body weight of C60 fullerene in olive oil multiple times via oral gavage [23].

**Figure 1:**
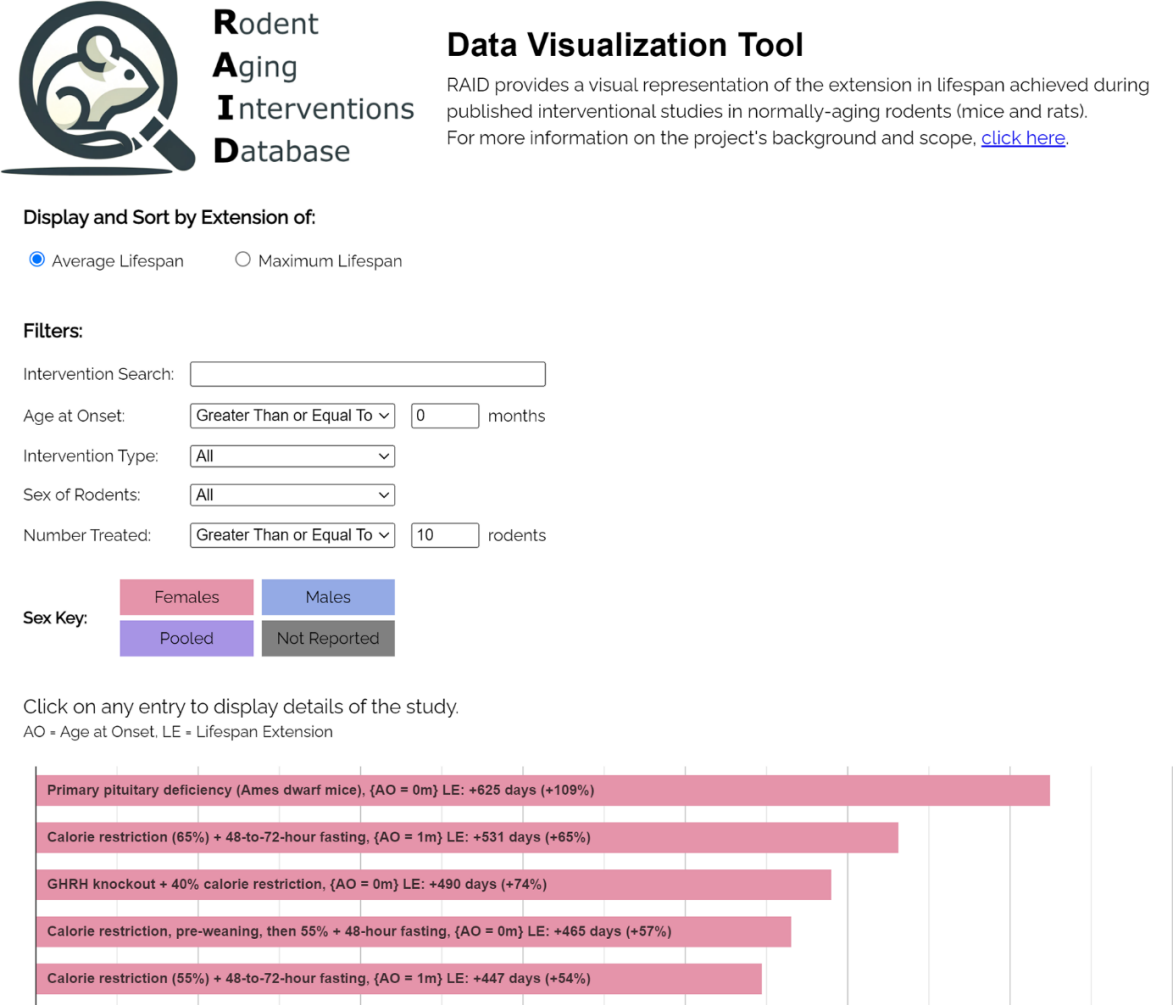
The RAID data visualization tool.

The RAID data visualization tool is publicly available at https://levf.org/raid. Figure 1 is an image of the tool in a web browser.

Our dataset yielded noteworthy observations. Firstly, omitting the as-yet-to-be-replicated C60 fullerene study, the five greatest median lifespan extension outcomes (+447 to +625 days) all occurred in females. Secondly, nine of the ten most effective interventions (+346 to +625 days) included either calorie restriction or interference with the GH/IGF-1 pathway (primary pituitary deficiency, GHRH knockout, and Pit1 mutation). Excluding the C60 fullerene report and studies using either calorie restriction or GH/IGF-1 interference, the three most effective interventions in each sex are presented in Table 1 and discussed thereafter.

**Table 1:** Three most effective interventions for each sex^a.^

	**Most effective**	**2^nd^ most effective**	**3^rd^ most effective**
**Females, mean/median**	+270 days with VEGF overexpression from birth	+239 days with 14.7 ppm rapamycin in food and 1,000 ppm acarbose in food starting at 9 months	+204 days with 14 ppm rapamycin in food and 1,000 ppm metformin in food starting at 9 months
**Females, maximum**	+300 days with VEGF overexpression from birth	+218 days 14.7 ppm rapamycin in food and 1,000 ppm acarbose in food starting at 9 months	+186 days with 14 ppm rapamycin in food and 1,000 ppm metformin in food starting at 9 months
**Males, mean/median**	+360 days with VEGF overexpression from birth	+263 days with 14.7 ppm rapamycin in food and 1,000 ppm acarbose in food starting at 9 months	+255 days with 24-hour fasting every other day for 3 days per week starting at 20 weeks
**Males, maximum**	+405 days with 10 g/L of N-acetylcysteine in drinking water starting at 7 months	+400 days with 5 g/L of N-acetylcysteine in drinking water starting at 7 months	+345 days with VEGF overexpression from birth

^a^ These are the interventions with the largest increase in lifespan (in days) in our dataset after excluding studies about calorie restriction, GH/IGF-1 interference, and the exceptional C60 fullerene study [23].

Referring to Table 1, note that overexpression of vascular endothelial growth factor (VEGF) ranks in the top three for both males *and* females in median lifespan extension and for males in maximum lifespan extension. Because this intervention may be operating via a different mechanism of action compared to more popularly discussed interventions (dietary restriction, GH/IGF-1 interference, metformin, rapamycin, and senolytics), we suspect VEGF overexpression may be particularly interesting for further research. Additionally, note that while N-acetylcysteine in drinking water was a high-ranking intervention in males for maximum lifespan extension, it had no significant effect on median or maximum lifespan in females [25]. This suggests that in late life, male—and not female—mouse lifespan may be limited by redox-related degeneration that is substantially alleviated by dietary N-acetylcysteine, and that most of the positive effects might be achieved at a dose of 5 g/L of drinking water or lower. Finally, we note that rapamycin with acarbose was among the top three interventions for both male and female mice, suggesting that this combination may have significant and consistent benefits regardless of sex.

Unfortunately, key data and statistical information were missing from a substantial number of our selected studies. For example, 11 of 121 studies (9%) appeared not to report the number of rodents in the treatment group, and 21 of 116 (18%) did not report specific p-values for their findings on mean/median lifespan. Approximately 25% of studies did not explicitly report the numerical value for lifespan (in days) for treatment and control groups, and those figures had to be estimated from survival curves. The number and proportion of studies apparently missing essential data are reported in Table 2.

**Table 2:** Number and proportion of studies missing essential information.

**Statistical parameter**	**Studies missing (#)**	**Studies missing (%)**
# of controls	12/121	9.9%
# treated	11/121	9.0%
p-value for mean/median lifespan	21/116^a^	18.1%^a^
p-value for maximum lifespan	62/105^b^	59.0%^b^
Definition of maximum lifespan	35/107^b^	32.7%^b^
Explicit report of lifespan (in days)^c^	30/121	24.7%

^a^The denominator is the number of studies which reported an effect on mean/median lifespan in the treatment group relative to controls. (Studies reporting no effect on this parameter were excluded from this calculation.)

^b^The denominator is the number of studies which reported an effect on maximum lifespan in the treatment group relative to controls. (Studies reporting no effect were excluded from this calculation.) The two denominators differ (105 vs. 107) because some studies gave a definition for maximum lifespan but did not report a significant effect on it, and those were excluded from the denominator for the studies expected to report p-values for maximum lifespan extension.

^c^This refers to studies for which numerical lifespan data was not reported in the text and estimation was required from the reported survival curve(s).

## DISCUSSION

Our original data table contains primary data from selected studies published through October 2022. We intend to periodically update our dataset by (1) using refined and expanded PubMed queries to identify studies that our initial queries missed and studies that have been published after October 2022, (2) using machine learning algorithms trained with our initial dataset to identify other candidate studies in the literature, and (3) considering studies nominated by others. If readers have reviewed our RAID visualization tool in detail (including the list of pending inclusions provided at https://www.levf.org/projects/raid#about) and are aware of a relevant study that we appear to have missed, we invite them to nominate that study for inclusion by emailing us at raid@levf.org.

Future additions to our dataset will also include data from studies which investigated a possible life-extension effect in rodents but failed to observe one or observed negative effects. Furthermore, data from studies using other rodents such as guinea pigs, gerbils, squirrels, chipmunks, and naked mole rats would also be appropriate for RAID, but we did not perform targeted queries for these species for the version reported here. If other investigators are interested in collaborating with us to expand the dataset, we invite them to email us at raid@levf.org.

Given that our current dataset is based on searches extending only up to October of 2022, we did consider bringing the collection fully up to date (using the original search strategies described herein) before submitting this manuscript for publication. However, we are also working on improvements to our data collection methods, which we plan to implement in our next batch update. Particularly, we are in the process of developing machine learning algorithms to identify relevant studies for RAID, using our existing dataset as true positives to train software to identify other candidate studies for inclusion. We intend to use these algorithms to search both PubMed and the wider Internet for relevant studies. This is in addition to the new PubMed query phrases we have developed since our initial screen, based in part on analysis of the existing dataset. These methods will be applied not only to the years 2022-2025 but to all publication dates. We expect these techniques to substantially expand our dataset, but as they will require significant additional time and effort to implement, we intend to introduce them in a batch update to RAID before the end of 2025. After that point, maintaining RAID’s currentness should require relatively little time investment.

Missing data significantly limited the types of evaluation which could be conducted. For example, the web-based data visualization tool does not include the ability to filter by p-values, since nearly 20% of median lifespan data and nearly 60% of maximum lifespan comparisons were not accompanied by p-values. Relatedly, a fundamental caveat about our dataset is that approximately 25% of lifespan figures in these studies had to be manually estimated from survival curves because exact data could not be found in the associated manuscripts.

To enhance meta-analysis of lifespan extension studies and promote efficient data collection, we humbly suggest that investigators provide a minimum set of essential study parameters and statistical information in all future publications. Data aggregation and summary analysis is more efficient when these parameters are published clearly and precisely. Furthermore, given advancing machine intelligence, access to this data in an easily searchable format will allow for new methods of aggregation and analysis. We present our suggestions for minimum essential data for all future rodent life extension investigations in Table 3.

**Table 3:** Suggested minimum essential data for rodent life-extension investigations.

**Study parameters**	**Lifespan effects**	**Statistical information**
Species Strain Sex Genetic modifications Intervention, dose, and route of administration Age at initiation of intervention Numbers of living, uncensored rodents in all control and treatment groups at each point of comparison	Lifespan (in days) at 90% survival, 50% survival, and 10% survival, for control and treatment groups Survival curves of each treatment group and its control group in a single figure	Specific p-values for comparisons of lifespan at 90% survival, 50% survival, and 10% survival, for treatment groups relative to controls, regardless of significance The type of statistical test used to assess the above comparisons (e.g., Fisher’s exact test, Boschloo’s test) Definition of maximum lifespan if something other than lifespan at 10% survival is used (e.g., average lifespan of last 2 rodents) The logrank test and its associated p-value for the comparison of the survival curves of the treatment groups compared to their control groups.
Publish raw lifespan data: Lifespan (in days) of each mouse in all control and treatment groups (e.g. in a supplementary data file), noting any animals lost (censored) from the study due to non-age-related causes (accidents, fighting, etc.)		

Most of the parameters in Table 3 were usually reported in the studies in our dataset. However, we wish to emphasize a few noteworthy recommendations here. First, we recommend reporting the individual lifespans of all mice in all treatment and control groups, probably in a supplementary data file. This will enable other groups to perform extended analyses on the study findings. Second, to assess the effects of an intervention on early mortality, we recommend henceforth including the comparison of the lifespans at 90% survival between treatment and control groups. Historically, an assessment of the effect of an intervention on early mortality has not commonly been reported. When combined with lifespan at 50% survival (median) and 10% survival (maximum), these comparisons assess the effects of an intervention on the lifespan at different life stages, and the presence of an effect on one life stage without an effect on the others can offer potentially useful insights. One example is the effect of N-acetylcysteine affecting late-life mortality in males and not females, discussed earlier. Another example is one group of investigators in our dataset who failed to find a significant benefit of an intervention on median or maximum lifespan but reported one on mean lifespan. It was apparent from the survival curve that early-life mortality was reduced by this intervention, but that effect may have been overlooked had the investigators only evaluated median and maximum lifespan [26]. Because mean lifespan is affected more by outliers than the other parameters, and because it does not indicate which stage of life was affected by the intervention, we recommend against using it.

The age at which 90% of rodents are still alive may be a useful measure of the effect of an intervention on early-life mortality. This is the point in the life cycle of common lab mouse strains when the mortality rate is accelerating but has not yet reached its peak. Thus, a delay in the time until a cohort reaches 10% mortality will suggest that early mortality has been delayed by an intervention. It is perhaps debatable whether lifespan at 90% survival is the optimal point to assess early mortality in rodents—the exact figure might be decided among experts in the field. But it is clearly a more specific indicator of early-life mortality than mean lifespan. Assessments of mid-life and late-life mortality (lifespans at 50% and 10% survival, respectively) have been routine in the literature, and we have no modifications to suggest for assessing effects on mortality at these stages of the life cycle.

Additionally, we suggest reporting these lifespan data with their exact p-values regardless of statistical significance. Routinely doing so may help normalize reporting results of intervention protocols having p-values above 0.05 and thereby encourage the reporting of studies finding no significant effects, which can also be informative. Moreover, consistent reporting of these data will enable sorting of aggregated results according to p-values. This capability might be of interest to investigators who want to choose the interventions with the highest likelihood of positively affecting their intervention groups (interventions with the lowest p-values) or who want to attempt to re-test an intervention that displayed questionable effectiveness (interventions with p-values close to 0.05).

**Figure 2:**
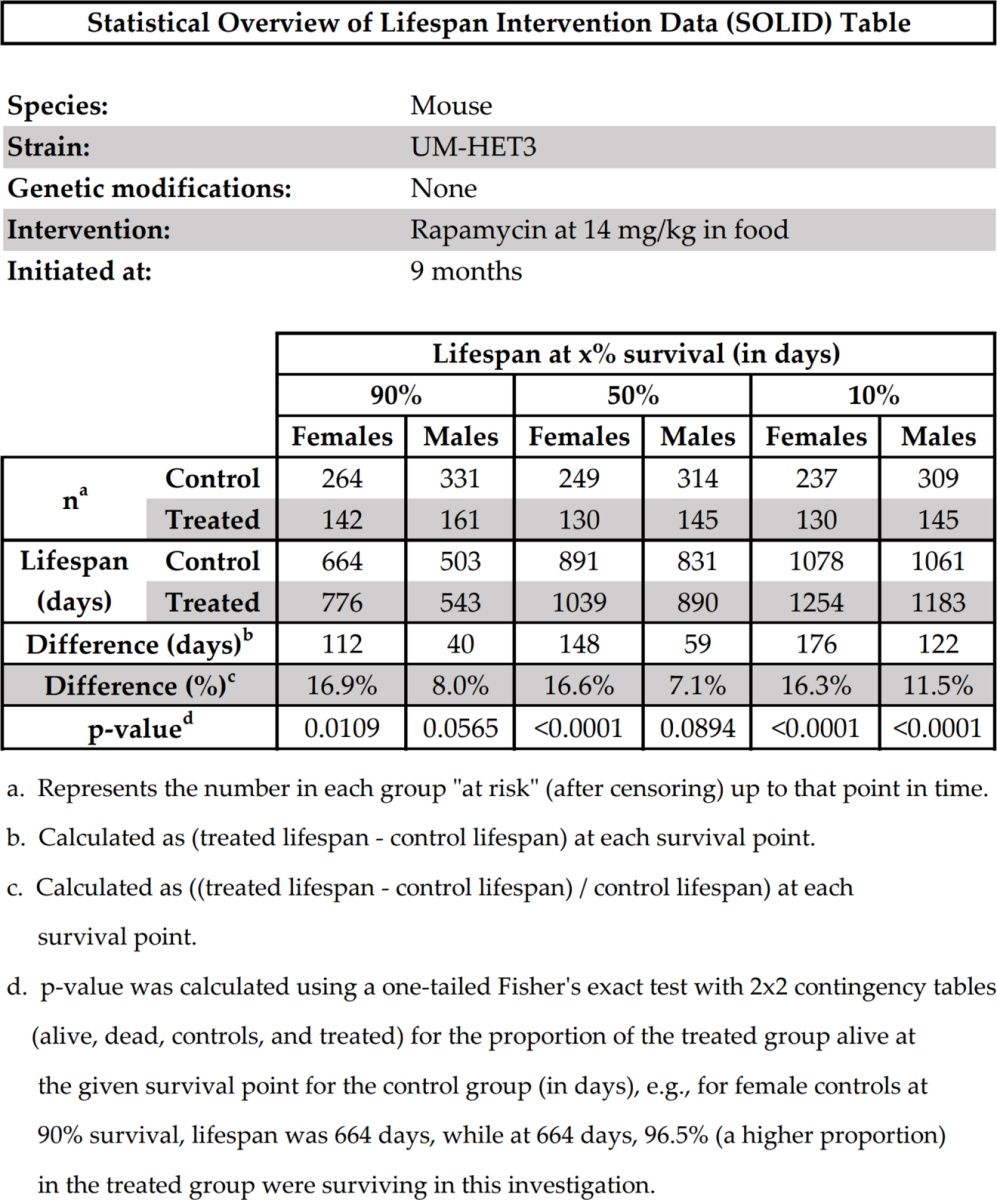
Statistical overview of lifespan intervention data (SOLID) table.

To illustrate what most of our recommendations might look like if they were efficiently presented in a single table, we present Figure 2, which we call the “Statistical Overview of Lifespan Intervention Data (SOLID) Table”. To create this table, we used the raw data from the investigation of the effects of rapamycin on mouse lifespan by [27]. We invite other researchers to consider using this table’s format to summarize their future investigations.

A copy of the original SOLID Table in Microsoft Excel is provided in the Supplementary Data File for other investigators to use as a template for reporting their own findings in this format.

Reliable access to these data in a standardized format across studies can facilitate rapid aggregation and comparison of many different interventions to evaluate the stage(s) of life at which each intervention affected mortality. We are currently interested in collaborating with the authors of the studies in our dataset to obtain their primary study data so that our full set of data recommendations can be added to their respective entries in RAID.

Finally, we have made our primary data table containing all RAID entries available with this manuscript. It can be found in the Supplementary Data File.

## MATERIALS AND METHODS

Our methods can be classified into two main project areas: (1) PubMed queries and data extraction, and (2) construction of our web-based data visualization tool.

### PubMed query design

All studies included in RAID had two main sources: (1) the PubMed database located at https://pubmed.ncbi.nlm.nih.gov/, and (2) nomination by our colleagues.

Our PubMed queries were designed to successfully identify a high proportion of studies reporting a positive effect on the lifespans of mice or rats relative to controls. Unusually short-lived strains were excluded, including strains used as disease models. Because the choice of words and phrases used to describe life-extension investigations in the literature has varied considerably between investigators, our PubMed queries had to be complex enough to account for this variability.

In designing our PubMed queries, we first combined synonyms for the rodents of interest (mice and rats) into a single query string. For this investigation, our PubMed query string was:

*(mice[tiab] OR mouse[tiab] OR mus[tiab] OR musculus[tiab] OR murine[tiab] OR rat[tiab] OR Rattus[tiab] OR norvegicus[tiab] OR rodent[tiab])*

(Note that “[tiab]” is the PubMed field tag for querying only the title and abstract fields of PubMed entries.)

This query string was then combined (using the “AND” operator) with other query strings which used synonyms for the word “lifespan”, such as “life-span”, “healthspan”, “health-span”, “longevity”, and others, resulting in a compound query which was then submitted to PubMed. Manual screening of the results followed, as described below.

Each study in the search results was evaluated for relevance according to its title. Retained studies were then evaluated based on their abstracts. Studies with an abstract suggesting a high likelihood of being relevant were retained for evaluation of their full-text manuscripts. The manuscripts were then read to confirm their relevance, rejected if irrelevant, and their essential data extracted if relevant. Extracted data was aggregated into the spreadsheet upon which the web-based data visualization tool was based. Not all studies reported mean, median, and maximum lifespan data, but most studies reported either mean or median lifespan data, and most reported maximum lifespan data.

### Data extraction

The data extracted from PubMed and the full-text manuscripts included the PubMed ID (PMID) of the study, the study title and bibliographic information, the intervention reported to be associated with rodent life extension, the type of rodent (mouse or rat), the strain of rodent, the sex of the group in which the effect was observed, the type of intervention (genetic, pharmaceutical, surgical, etc.), the number of rodents in the control and treatment groups, the mean or median lifespans (in days) of the control and treatment groups, the percentage difference between the mean/median lifespans of the control and treatment groups, the p-values for the differences in mean/median lifespan between those groups, the definition of maximum lifespan used by the investigators (if reported), the maximum lifespans of the control and treatment groups, the percentage difference between the maximum lifespans of the control and treatment groups, the p-value associated with the difference in maximum lifespans, and special notes about the study. This data was recorded in a spreadsheet with each row describing the comparison between one intervention group and its control group. Note that approximately 25% of the studies in our dataset apparently did not report exact lifespan data, so it was manually estimated from survival curves in those manuscripts. These estimations may be slightly different than the actual data.

### Construction of the web-based data visualization tool

The web-based data visualization tool was written in JavaScript and can be accessed at the URL https://levf.org/raid. Upon navigating to this URL, the software tool loads the entire dataset from the data spreadsheet into the client browser, which enables rapid searching/filtering without further network requests. The software tool visualizes each row of the data spreadsheet as a horizontal bar, with the length of the bar being commensurate with the magnitude of lifespan extension reported in the associated study. Bars are sorted in descending order based on the number of days of lifespan extension reported in each respective intervention group relative to its control group and are color-coded according to the sex of the groups of rodents for convenient visualization based on sex. As a default setting, the bar chart is sorted according to the mean/median lifespan extension, but the user has the option to sort it based on the increase in maximum lifespan extension (in days).

Filters are provided to constrain the subset of the data displayed in the bar chart. Filters can be applied to the: (1) name of the intervention (case-insensitive substring match), (2) sex of the rodents, (3) age at administration, (4) type of intervention, and (5) number of rodents treated. Clicking any individual bar causes a table containing the study details (study title, PMID, authors, p-values, etc.) to drop down below the bar.

## Supplementary Material

Supplementary Data File 1
